# Genome-wide identification, phylogenetic and expression pattern analysis of MADS-box family genes in foxtail millet (*Setaria italica*)

**DOI:** 10.1038/s41598-022-07103-9

**Published:** 2022-03-23

**Authors:** Dili Lai, Jun Yan, Ailing He, Guoxing Xue, Hao Yang, Liang Feng, Xiaobao Wei, Long Li, Dabing Xiang, Jingjun Ruan, Yu Fan, Jianping Cheng

**Affiliations:** 1 College of Agriculture, Guizhou University, Huaxi District, Guiyang, 550025 Guizhou People’s Republic of China; 2 School of Food and Biological Engineering, Chengdu University, Longquanyi District, Chengdu, 610106 Sichuan People’s Republic of China; 3 Chengdu Institute for Food Control, Chengdu, 610030 People’s Republic of China; 4 Guizhou Provincial Center for Disease Control and Prevention, Guiyang, 550025 People’s Republic of China; 5 Henan University of Technology, Zhengzhou, 450001 People’s Republic of China

**Keywords:** Evolution, Genetics, Plant sciences

## Abstract

Foxtail millet (*Setaria italica*) is rich in nutrients and extremely beneficial to human health. We identified and comprehensively analyzed 89 MADS-box genes in the foxtail millet genome. According to the classification of MADS-box genes in *Arabidopsis thaliana* and rice, the *Si*MADS-box genes were divided into M-type (37) and MIKC-type (52). During evolution, the differentiation of MIKC-type MADS-box genes occurred before that of monocotyledons and dicotyledons. The *Si*MADS-box gene structure has undergone much differentiation, and the number of introns in the MIKC-type subfamily is much greater than that in the M-type subfamily. Analysis of gene duplication events revealed that MIKC-type MADS-box gene segmental duplication accounted for the vast majority of gene duplication events, and MIKC-type MADS-box genes played a major role in the amplification of *Si*MADS-box genes. Collinearity analysis showed highest collinearity between foxtail millet and maize MADS-box genes. Analysis of tissue-specific expression showed that *Si*MADS-box genes are highly expressed throughout the grain-filling process. Expression analysis of *Si*MADS-box genes under eight different abiotic stresses revealed many stress-tolerant genes, with induced expression of *SiMADS33* and *SiMADS78* under various stresses warranting further attention. Further, some SiMADS-box proteins may interact under external stress. This study provides insights for MADS-box gene mining and molecular breeding of foxtail millet in the future.

## Introduction

The MADS-box genes are divided into two categories: ARG80/SRF genes in animals and fungi, also known as M-type genes in plants; and MEF2 genes in animals and yeast, known as MIKC-type genes in plants^1^. The MADS genes of M-type are divided into three types: Mα, Mβ, and Mγ. And MIKC-type MADS genes are divided into two major categories, namely MIKC* and MIKC^C^. MIKC^C^ is further divided into 14 subfamily in rice: GLO-like, DEF-like, GGM13-like, OsMADS32-like, AGL12-like, STMADS11-like, AG-like, AGL17-like, AGL15-like, FLC-like, TM3-like, AGL6-like, AGL2-like and SQUA-like^1^. Serving as transcription factors, MADS-box genes are widely distributed in eukaryotes (plants, animals and fungi)^2^. Evolutionarily speaking, replication of the original MADS-box gene occurred before the differentiation of animals and plants^3^, indicating that the MADS-box gene family is relatively ancient. There are many reports on the functions of MADS-box genes, which regulate many growth processes, such as flower organ development^4^, embryo development^5^, fruit development^6,7^ and vegetative organ development^8^. For example, the discovery of the ABC genetic model explained how the combined functions of three types of genes (A, B and C) determine the characteristics of four floral organs^4^. The activity of organ-recognition genes B and C was found to be closely related to three MADS-box genes (SEP1–3)^9^. In recent years, MADS-box genes have also been thoroughly studied in other plants. Several key MADS-box genes of moso bamboo (*Phyllostachys edulis*) are involved in inflorescence development, and ectopic overexpression of *PeMADS5* in *Arabidopsis thaliana* leads to early flowering and abnormal flower phenotypes^10^. The MADS-box gene of pineapple is closely related to flower density, and some MADS-box genes are involved in CAM photosynthesis and are regulated by a biological clock^11^. Similarly, many MADS-box genes involved in floral organ and fruit development have also been found in tomato^12^. MADS-box genes even play an important role in tuber plant dormancy^13^. Thus, MADS-box genes have many different roles.

In around 6000 BC in northern China, foxtail millet (*Setaria italica*) was domesticated from *Setaria viridis*^14^ and, together with *Panicum miliaceum*, became the main food crop at that time^15,16^. Foxtail millet is rich in nutrients such as calcium, dietary fiber, polyphenols, fat and protein^17,18^, which promote human health. Moreover, foxtail millet is a drought-resistant plant, which has a certain reference to the stress adaptation of crops and serves as a model C4 plant for studies. MADS-box genes have scarcely been studied in C4 plants. However, whole-genome sequencing of a foxtail millet species^16,19^ enabled us to conduct an in-depth analysis of the MADS-box gene family in this plant.

Analyses of gene or protein families in foxtail millet include WRKY^20^, ZIP^21^, TPS^22^, SSPs^23^, and WD40^24^, but studies of the MADS-box gene family in this plant are incomplete. Moreover, how *Si*MADS-box genes participate in the growth and development of foxtail millet and in its response to stress is not clear. Therefore, in-depth research on the MADS-box gene family in millet is still needed, as it will provide great assistance for gene mining and molecular breeding. We identified 89 MADS-box genes from the whole genome of foxtail millet, and they were classified into subfamilies according to the MADS-box gene family classification in *A. thaliana*^25^ and rice^1^: 37 genes belonged to the M-type and 52 to the MIKC-type. We analyzed their gene structure, exon/intron distribution, conserved motifs, molecular weight (Mw), isoelectric point (pI) and subcellular localization, and gene duplication events were revealed. Homology between *Si*MADS-box genes and MADS-box genes of other species was assessed and a phylogenetic tree was constructed. Finally, the tissue-specific expression of 12 *Si*MADS-box genes and their responses to different abiotic stresses are discussed.

## Results

### Identification of MADS-box genes in foxtail millet

Two BLAST methods were used to identify 89 MADS-box genes in foxtail millet. The genes were named *SiMADS1*–*SiMADS89* according to their chromosomal positions. Their genetic characteristics, including coding sequence (CDS) length, protein molecular weight, pI and subcellular localization, were determined. Among the 89 *Si*MADS-box proteins, SiMADS7 and SiMADS74 were the smallest with only 60 amino acids, and the largest one (SiMADS21) had 483 amino acids. The molecular weight of the proteins ranged from 6.78 to 53.18 kDa, and pI ranged from 4.41 (SiMADS21) to 11.39 (SiMADS35). Predicted subcellular localization revealed 1 in the endoplasmic reticulum, 11 in the mitochondria, 19 in the chloroplast, 14 in the cytoplasm, and 44 in the nucleus (Table S1).

### Phylogenetic analysis and classification of the SiMADS-box genes

**Figure 1:**
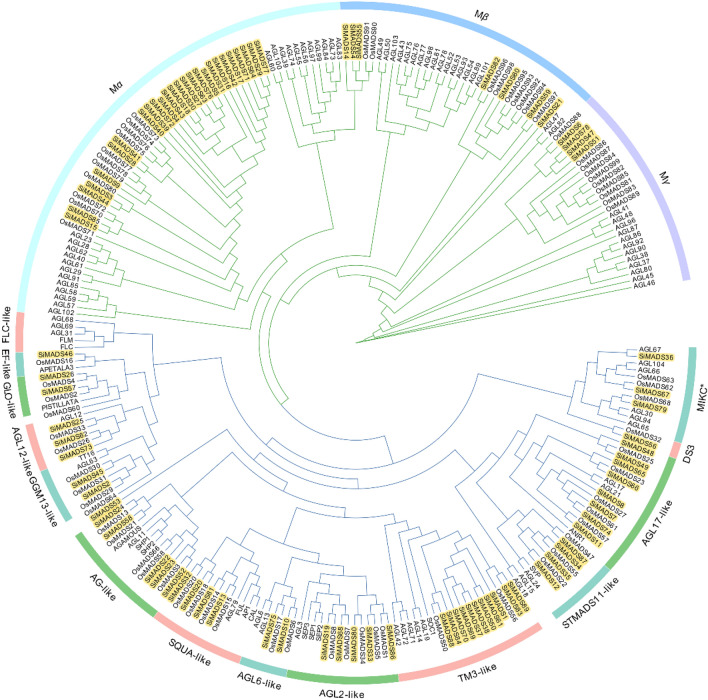
Unrooted phylogenetic tree showing relationships among MADS-box proteins of *Setaria italica*, rice and *Arabidopsis thaliana*. The phylogenetic tree was derived using the ML method in MEGA X. The tree shows the 17 phylogenetic subfamilies. MADS-box proteins from rice are marked with the prefix ‘Os’.

The phylogenetic relationships of the 89 *Si*MADS-box proteins was studied by multiple sequence alignment. MADS-box gene classification of *A. thaliana* and rice was referenced. The foxtail millet MADS-box phylogenetic tree classified the *Si*MADS-box genes into two subgroups: 37 M-type genes and 52 MIKC-type genes. The *Si*MADS-box genes were similar to those of the monocotyledon rice, and could be further divided into 17 groups: 14 in the MIKC-type gene subfamily, and 3 in the M-type gene subfamily (Fig. 1). Interestingly, the FLC-like subfamily did not contain *Si*MADS-box genes, in contrast to both *Arabidopsis*^25^ and rice^1^. We also investigated the number of MADS-box genes in maize^26^, sorghum^26^, rice^1^, *Arabidopsis*^25^ and *Brassica rapa*^27^. The numbers and proportions of MADS-box genes in each subfamily are shown in Table 1. C4 plants (foxtail millet, maize, sorghum) had similar proportions of MIKC-type MADS-box genes as C3 plants. However, there were significant differences between the M-type genes in C4 vs. C3 plants. The proportion of Mα subfamily members in C4 plants was much higher than in C3 plants. On the other hand, the proportion of Mβ and Mγ subfamily members in C4 plants was much lower than in C3 plants. These results suggest that after the divergence of C3 and C4 plants, perhaps more members of the Mα subfamily were retained by the C4 plants.

**Table 1:** The number and proportion of each type of MADS-box genes in foxtail millet, maize, sorghum, rice, *Arabidopsis* and *Brassica rapa.*

	Total	MIKC^C^		MIKC*		Mα		Mβ		Mγ	
		Number	Percentage	Number	Percentage	Number	Percentage	Number	Percentage	Number	Percentage
*S. italica*	89	49	55.06	3	3.37	26	29.21	7	7.87	4	4.49
Maize	75	39	52.00	4	5.33	27	36.00	3	4.00	2	2.67
Sorghum	65	33	50.77	2	3.08	26	40.00	2	3.08	2	3.08
Rice	75	39	52.00	4	5.33	13	17.33	9	12.00	10	13.33
Arabidopsis	108	39	36.11	8	7.41	25	23.15	20	18.52	16	14.81
*B. rapa*	167	89	53.29	16	9.58	11	6.59	29	17.37	22	13.17

Therefore, members of the Mα subfamily may have played a major role in the evolution of C4 plants.

### Gene structure, motif composition, and protein-interaction predictions for the *Si*MADS-box gene family

**Figure 2:**
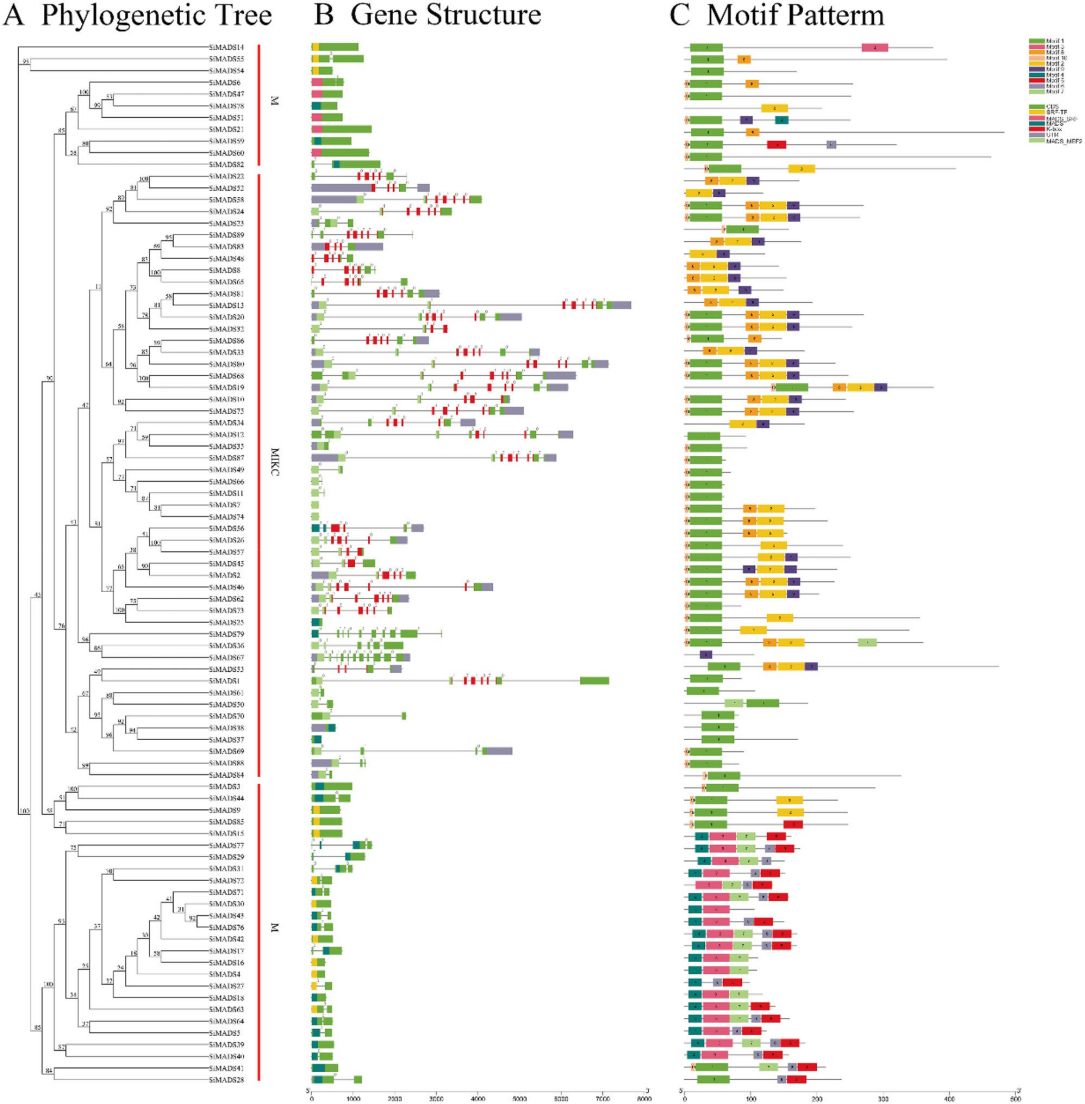
Phylogenetic relationships, gene structure and architecture of the conserved protein motifs in 89 genes from *S. italica*. (**A**) The phylogenetic tree was constructed based on the full-length sequences of *S. italica* MADS-box proteins. (**B**) Exon–intron structure of *S. italica* MADS-box genes. Lines represent introns, boxes represent exons, and domains are color-coded. Number indicates the phase of the corresponding intron. (**C**) Amino acid motifs in the *Si*MADS-box proteins (1–10) are represented by colored boxes. Black lines indicate relative protein lengths. Sequence information for each motif is provided in Table S2.

Introns are ubiquitous in eukaryotes, which is an important feature different from prokaryotes. In higher organisms, introns have been reported to regulate gene expression at multiple levels. The main function of introns is to generate different exon combinations through differential splicing to translate different proteins, which improves the complexity of the proteome^28,29^. There are two arguments about the origin of introns, namely the hypotheses of early introns and late introns, which are still inconclusive at present. However, more researches tend to the early intron hypothesis, that is, there are a large number of introns in relatively old ancestors, which means that the loss of a large number of introns may be common in eukaryotic evolution, and the acquisition of a large number of introns may be rare^30–33^. The latest research also shows that introns themselves have important functions independent of their coding genes. Introns can mediate the cell's response to starvation^34^, and it can also regulate the growth rate under stress conditions and improve the adaptability of yeast^35^. Therefore, introns are very important to organisms, so we also analyzed the differences in introns of two types of foxtail millet MADS genes. Exon and intron structures of *Si*MADS-box genes were obtained by comparing their CDSs with the corresponding genomic DNA sequences. In general, the exon/intron structures of different members of the same subfamily should be similar. As shown in Fig. 2, the introns of *Si*MADS-box genes ranged from 0 to 10. The mean number of introns in the MIKC-type subfamily was about 4.3, and that in the M-type subfamily was about 0.6, suggests that M-type gene has undergone great differentiation. In the M-type subfamily, the lowest average number of intron, 0.25, was found for the Mγ branch. Among the 37 M-type genes, 19 (51.35%) had no intron structure. This may be further evidence for significantly different rates of evolution for the M-type branches. However, the average number of introns for the MIKC* branch of the MIKC-type subfamily was largest, as high as 8. The foxtail millet MADS-box genes were found to contain mainly the MADS, MADS-SRF, MADS-MEF2 and K-box domains.

The online MEME program was used to analyze the motifs of the 89 *Si*MADS-box proteins, and a structural diagram of these proteins was constructed. We identified 10 conserved motifs (Table S2). The motif composition of *Si*MADS-box proteins in the same subfamily was similar, and differed in different subfamilies. As can be seen from Fig. 2, the motif order for the MIKC-type subfamily was mainly 10–1–8–2–9, and these motifs could be conserved. The conserved motif order for the Mα subfamily was suggested to be 4–3–7–6–5, where the differences in the conserved motif patterns might be related to the proteins' specific functions, but this requires further elucidation.

**Figure 3:**
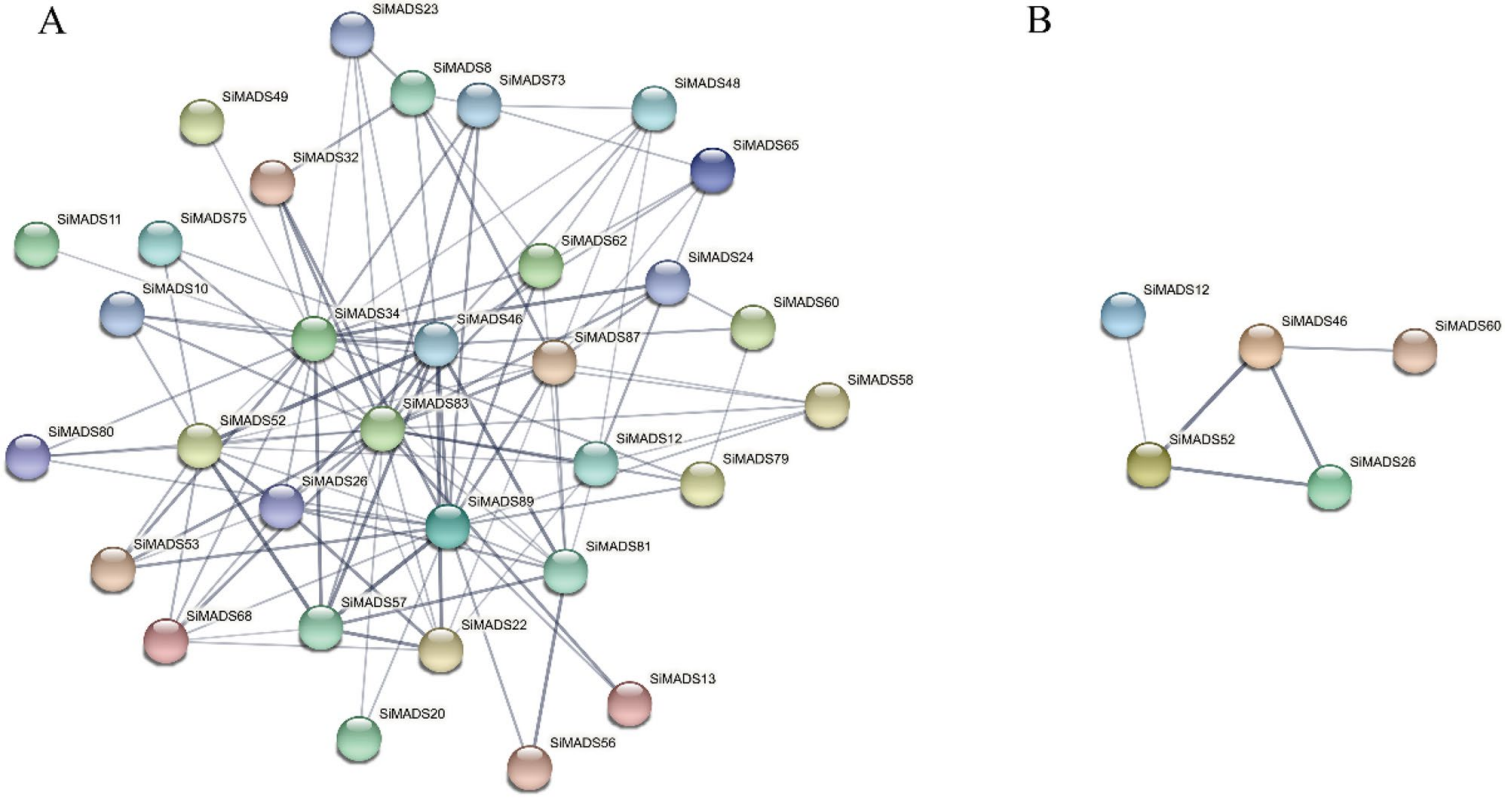
Predicted interactions between foxtail millet MADS-box proteins. (**A**) Prediction of the protein–protein interaction network among 89 SiMADS. (**B**) Prediction of the protein–protein interaction network among 5 SiMADS in stress treatments.

These results showed that motif sequences patterns in a same subfamily are basically similar, which may also indicate that these proteins have similar functions or participate together in some pathways. We therefore turned to STRING^36^ to predict interactions among the 89 *Si*MADS-box proteins. The results are shown in Fig. 3. Among the 89 *Si*MAD-box proteins, 32 were predicted to interact with each other. Among these putatively interacting proteins, 31 were MIKC-type, and only 1 (SiMADS60) was M-type. The higher number of interactions predicted for the MIKC-type MADS-box proteins might be related to their function.

### Chromosomal distribution and synteny analysis of the *Si*MADS-box genes

**Figure 4:**
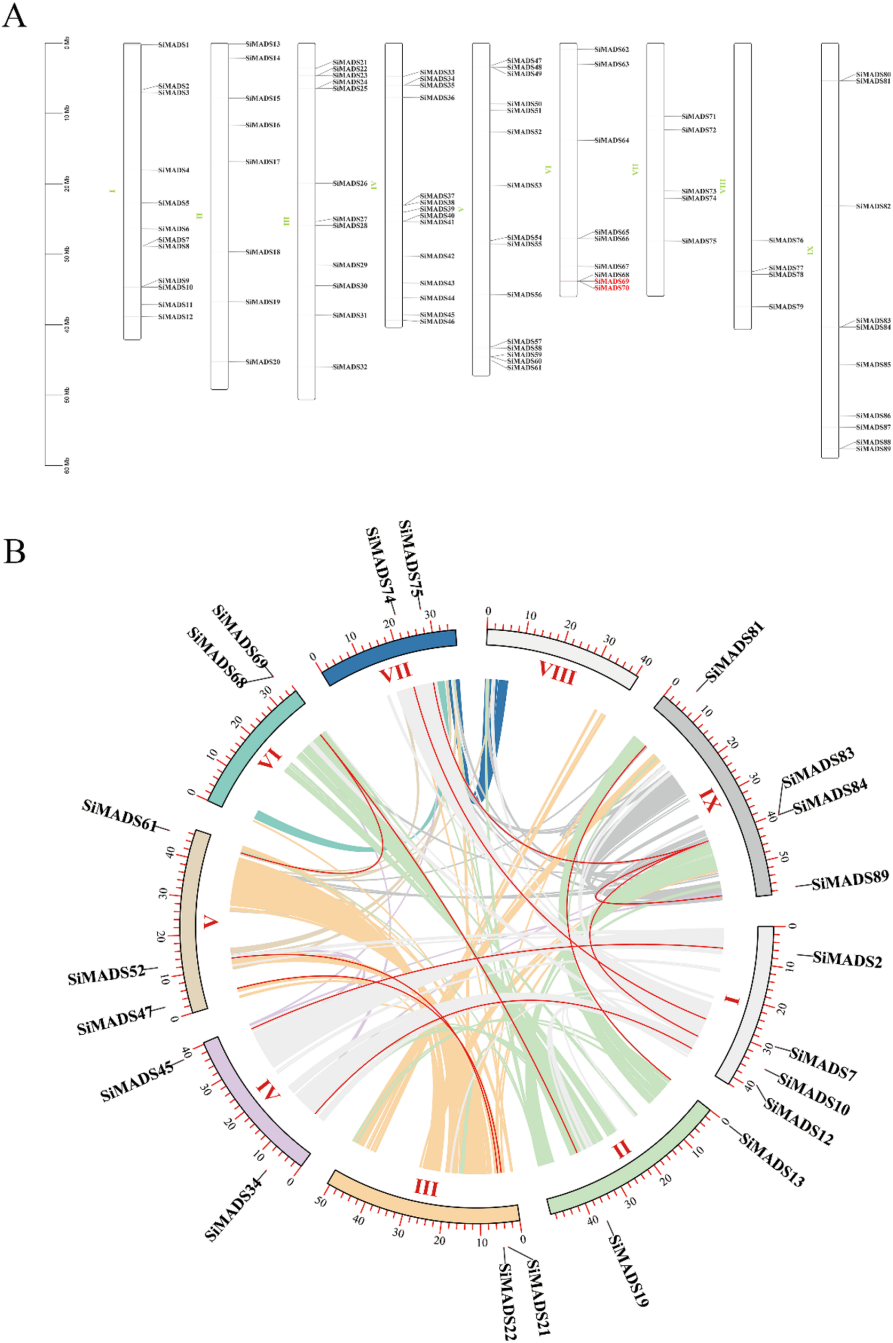
The chromosomal distribution and synteny blocks of the *S. italica* MADS-box genes. (**A**). Schematic representation of the chromosomal distribution of the *S. italica* MADS-box genes. Vertical bars represent the chromosomes of *S. italica*. The chromosome number is indicated to the left of each chromosome. The scale on the left represents chromosome length. (**B**). Schematic representation of the chromosomal distribution and interchromosomal relationships of *S. italica* MADS-box genes. Colored lines indicate all synteny blocks in the *S. italica* genome and red lines indicate duplicated MADS-box gene pairs. Chromosome number is indicated at the bottom of each chromosome.

The chromosomal positions of the *Si*MADS-box genes are shown in Fig. 4A. Chromosome VIII had the least number of MADS-box genes (4), and chromosome V, the most (15). Referring to the closely related genes in *A. thaliana* falling into each other in 200 kB as tandem duplicates^37^, only one pair of tandem duplicates (*SiMADS69* and *SiMADS70*) were found among the foxtail millet MADS-box genes (Fig. 4A). The number of linked genes in linkage group (LG) III/V was higher than that in other linkage groups, whereas the distribution of *Si*MADS-box genes was largest in LG IX (5). There were 11 pairs of segment duplicates among *Si*MADS-box genes (Fig. 4B, Table S3), many more than the number of tandem duplicates. Meanwhile, only one of the 11 pairs of segment duplicates belonged to M-type MADS-box gene, while the rest belonged to MIKC MADS-box gene (Table S3, Table 1). This may further explain why this is the largest branch of the foxtail millet MADS-box gene family, with the highest number of genes (49) and the highest proportion (55.06%) in each subfamily. Moreover, the proportion of this subfamily was higher than that in other species, even sorghum and corn, which are also C4 plants (Table 1). At the same time, the analysis of MADS-box gene structure also found that the MIKC^C^ branch, which has a large number of introns. These results suggest that some *Si*MADS-box genes may have been generated by gene-replication events, which may have been the main driving force for *Si*MADS-box gene evolution.

**Figure 5:**
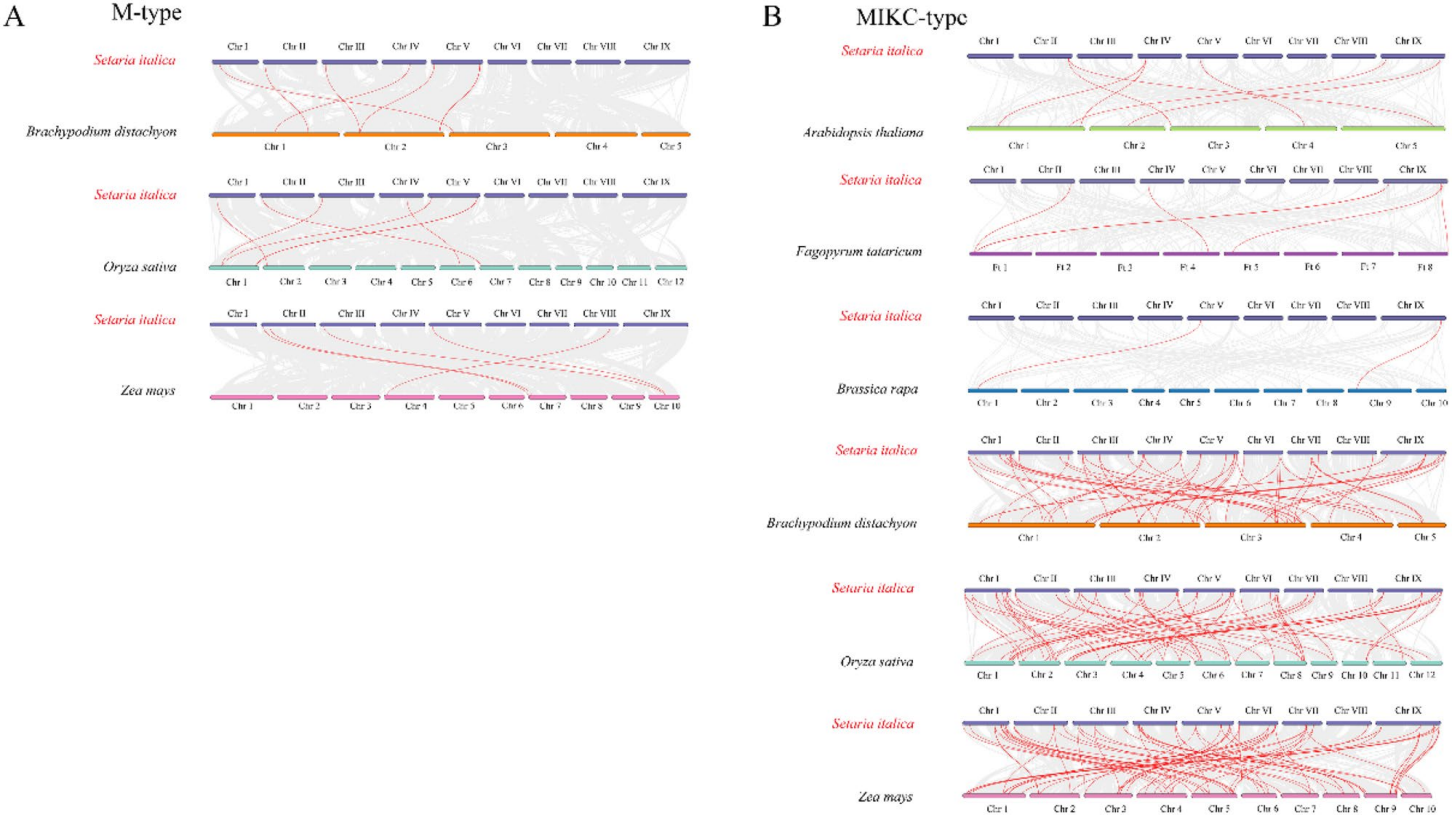
Synteny analysis of MADS-box genes between *S. italica* and other plant species. (**A**) Synteny analysis of the M-type MADS-box genes between *S. italica* and three representative plant species (*B. distachyon*, *Oryza sativa*, *Zea mays*). (**B**) Synteny analysis of the MIKC-type MADS-box genes between *S. italica* and six representative plant species (*A. thaliana*, *F. tataricum*, *B. rapa*, *B. distachyon*, *O. sativa*, *Z. mays*). Gray lines in the background indicate the collinear blocks within *S. italica* and other plant genomes, while red lines highlight the syntenic *MADS* gene pairs.

Collinearity analysis was conducted between M-type *Si*MADS-box genes and other plants (*A. thaliana*, *Brassica rapa*, *Fagopyrum tataricum*, *Brachypodium distachyon*, rice, and maize) (Fig. 5). The three dicotyledonous plants showed no collinearity with M-type *Si*MADS-box genes. There were only a few collinear genes between the M-type genes in foxtail millet, and those in *B. distachyon*, rice, and maize (Fig. 5, Table S4). Analysis of the collinearity map between MIKC-type *Si*MADS-box genes and those of other plants revealed highest collinearity with maize (71), followed by rice (62), *B. distachyon* (53), *A. thaliana* (7), *F. tataricum* (6) and *B. rapa* (2). Further analysis of these collinear genes revealed that seven MIKC-type *Si*MADS-box genes (*SiMADS20*, *SiMADS36*, *SiMADS52*, *SiMADS80*, *SiMADS81*, *SiMADS86*, and *SiMADS87*) exist in both monocotyledons and dicotyledons. Among these seven genes, only two (*SiMADS52* and *SiMADS81*) were included in the genes with segmental duplications. In addition, two MIKC-type genes (*SiMADS35*, *SiMADS66*) and two M-type genes (*SiMADS15*, *SiMADS78*) showed collinearity only with C4 plants (maize). In general, foxtail millet exhibited the highest collinearity with maize, suggesting that these C4 plants may have a close genetic relationship. In addition, for both M-type and MIKC-type *Si*MADS-box genes, the number of genes with collinearity to the monocots was much higher than that with collinearity to the dicots.

### Evolutionary analysis of MADS-box proteins from foxtail millet and several other species

**Figure 6:**
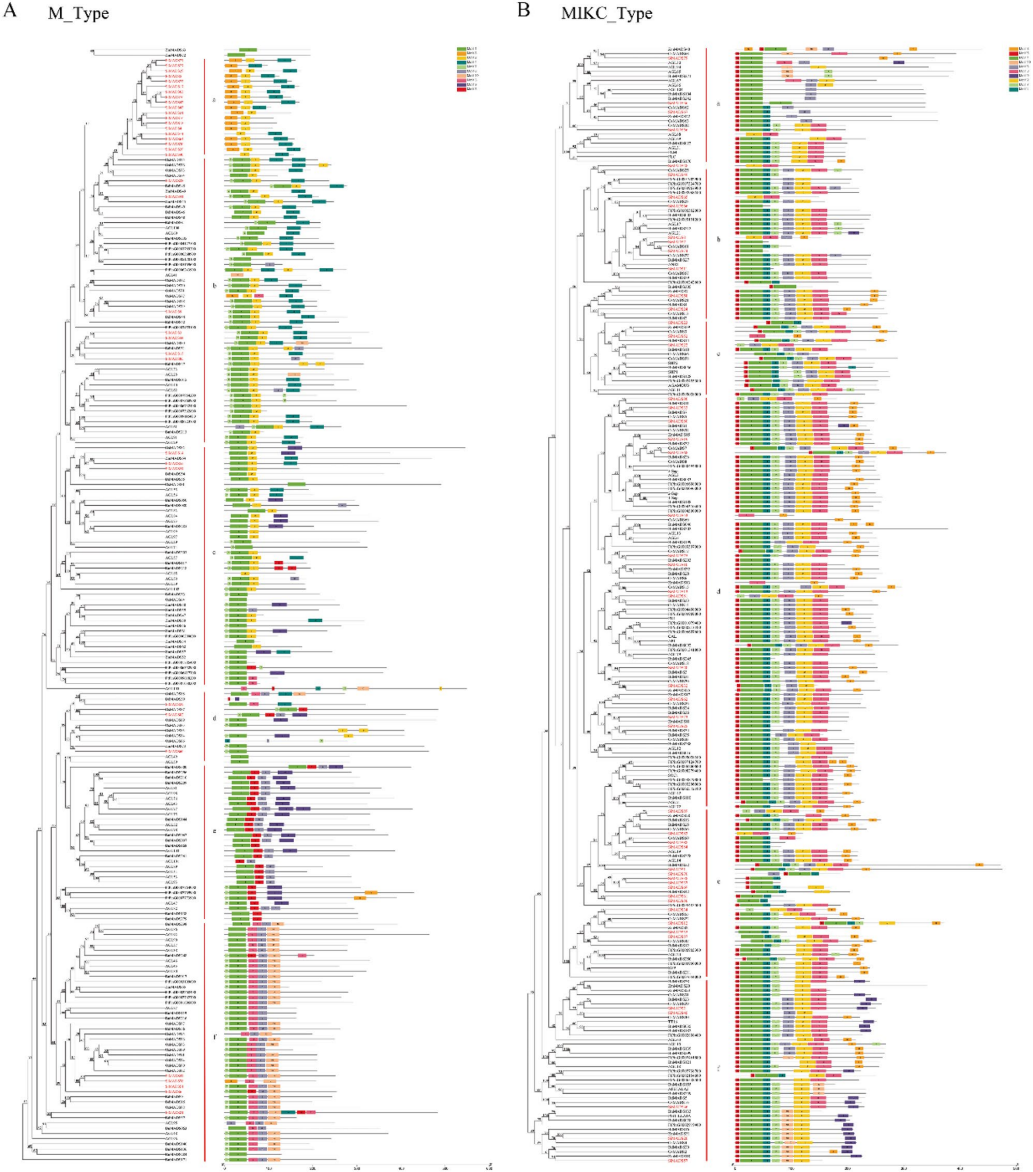
Phylogenetic relationship and motif composition of MADS-box proteins from *S. italica* with six different plant species (*A. thaliana*, *F. tataricum*, *B. napus*, *B. distachyon*, *O. sativa*, *Z. mays*). (**A**) Evolutionary relationship between M-type MADS-box proteins and motif composition. (**B**) Evolutionary relationship between MIKC-type MADS-box proteins and motif composition. Different-colored boxes represent different motifs and their positions in each MADS-box protein sequence. Sequence information for each motif is provided in Table S2.

We investigated the evolutionary relationship of M-type MADS-box proteins in the monocotyledons rice (31 genes), *B. distachyon* (17 genes) and maize (17 genes), and the dicotyledons *A. thaliana* (55 genes), *F. tataricum* (26 genes) and *Brassica napus* (30 genes) (Fig. 6A, Table S5). According to the phylogenetic tree, the M-type MADS-box proteins could be divided into six subfamilies, labeled a–f. The motifs of the M-type MADS-box proteins were analyzed by online MEME analysis. Motifs 7, 1 and 2 were conserved and distributed almost alternately in the whole subfamily. However, there were large differences among subfamilies. Subfamily a had the largest distribution of *Si*MADS-box members (19); their conserved motif order was 8–2–4, but they lacked motif 7–1. The main order of the b and c subfamily motifs was 7–1–2–4. The motifs of subfamily d were mainly 7–1, and subfamily e did not contain any *Si*MADS-box members. The main order of the motif of subfamily f was 7–1–3–6–10.

To explore the evolutionary relationship between MIKC-type MADS-box proteins of foxtail millet and the monocotyledonous plants rice (38 genes), *B. distachyon* (29 genes) and maize (16 genes), and dicotyledonous plants *A. thaliana* (43 genes), (38 genes), and *B. napus* (33 genes), we constructed a phylogenetic tree (Fig. 6B). There were six subfamilies (a–f) in the MIKC-type MADS-box family. Compared to the M-type MADS-box proteins, the motif distribution of the MIKC subfamily was more conservative. Its motif appeared most frequently in the order 5–1–4–7–6–2–3, and it was distributed in almost all subfamilies. However, there were also differences in some subfamilies, in particular subfamily f where motif 6 seemed to be replaced by 10. In addition, there was a new motif 9, which may confer unique physiological functions on this subfamily.

### Expression patterns of the *Si*MADS-box genes in different foxtail millet tissues

**Figure 7:**
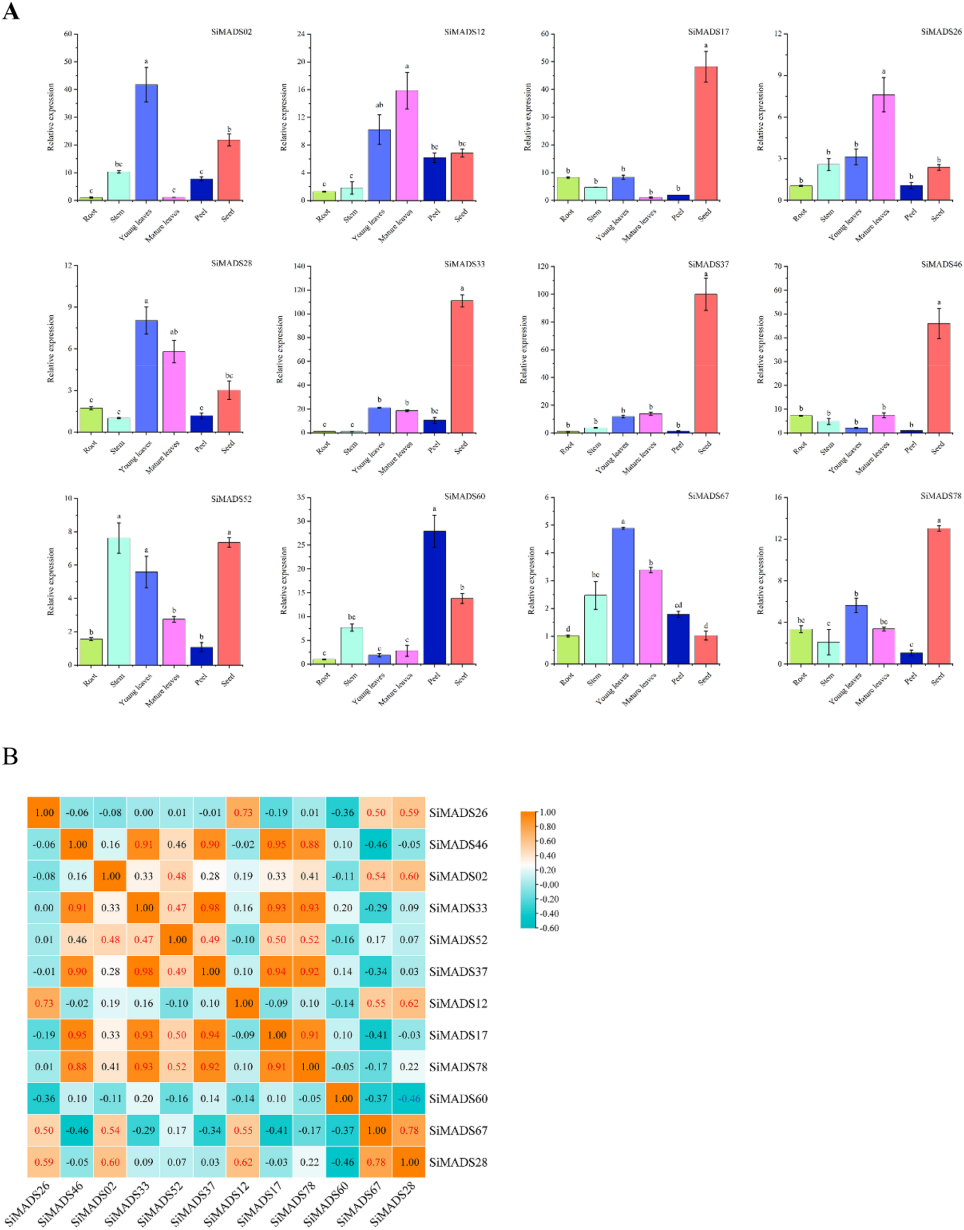
Tissue-specific expression of 12 *S. italica* MADS-box genes and their correlation with expression patterns in the middle stage of grain filling. (**A**) Expression patterns of 12 *S. italica* MADS-box genes in the root, stem, young leaves, mature leaves, peel and seed organs were examined by qRT-PCR. Error bars were obtained from three measurements. Lowercase letter above the bar indicates significant difference (α = 0.05, LSD) among treatments. (**B**) Positive number: positively correlated; negative number: negatively correlated. Red numbers indicate a significant correlation at the 0.05 level.

To study the physiological function of the *Si*MADS-box genes, the spatiotemporal expression of some members of the gene family was detected by qRT-PCR. Accumulation of eight MIKC-type and four M-type *Si*MADS-box gene transcripts in roots, stems, leaves (young leaves/mature leaves), peel and fruit in the middle stage of grain-filling was detected. Each of these genes belonged to a different subfamily. Since the function of MADS-box genes in floral organs is relatively clear, this study focused on the role of MADS-box genes in other tissues. These genes were expressed in all organs, but some were predominantly expressed in only a few tissues (Fig. 7A). Among them, *SiMADS17* and *SiMADS46* were highly expressed in roots, and *SiMADS02*, *SiMADS52* and *SiMADS60* were highly expressed in stems. In addition to *SiMADS46* and *SiMADS60*, other genes were highly expressed in young leaves. Six genes (*SiMADS12*, *SiMADS26*, *SiMADS28*, *SiMADS33*, *SiMADS37*, *SiMADS46*) were highly expressed in mature leaves. Four genes (*SiMADS02*, *SiMADS12*, *SiMADS33*, *SiMADS60*) were highly expressed in the peel. All genes except *SiMADS67* were highly expressed in the fruit, and in particular, the relative expression values of MIKC-type genes *SiMADS33* and *SiMADS37* were extremely high. These results, exhibiting differential expression patterns of *Si*MADS-box genes in different tissues of foxtail millet, indicated that the *Si*MADS-box genes have multiple functions in foxtail millet growth and development.

We also examined the correlation between *Si*MADS-box gene expression patterns in foxtail millet root, stem, leaf, peel and fruit; most of the genes were positively correlated (Fig. 7B). Expression of the M-type gene *SiMADS28* and MIKC-type gene *SiMADS67* was significantly positively correlated, and their expression was also significantly positively correlated with MIKC-type genes *SiMADS2*, *SiMADS12* and *SiMADS26*. These results indicated similar functions for M-type and MIKC-type *Si*MADS-box genes during plant growth and development. For example, expression of the M-type genes *SiMADS17* and *SiMADS78* showed a significant positive correlation, and their expression was also significantly positively correlated with MIKC-type genes *SiMADS33*, *SiMADS37*, *SiMADS46* and *SiMADS52*, which were strongly expressed in fruit.

### Expression patterns of MADS-box genes in foxtail millet during fruit development

**Figure 8:**
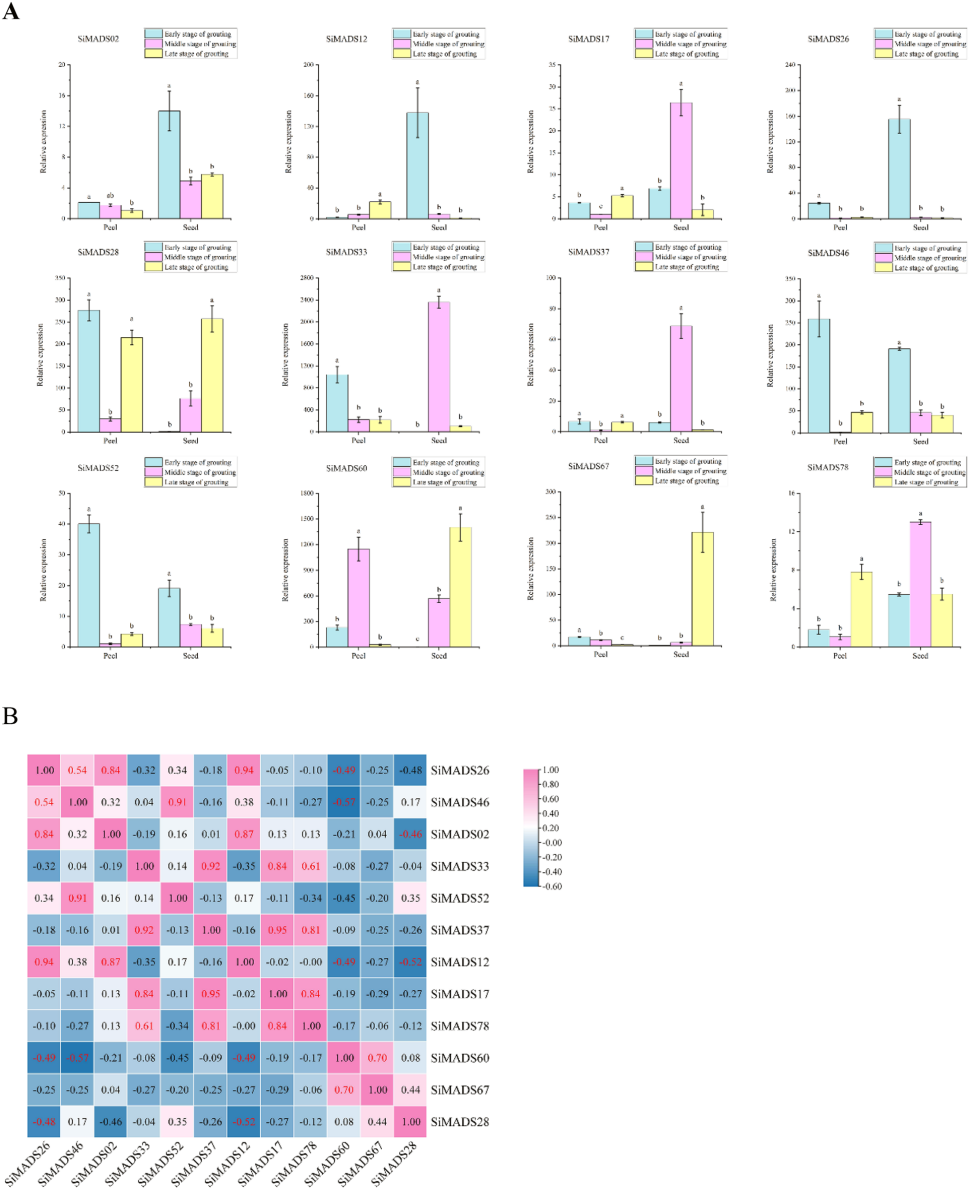
Expression pattern and correlation of 12 *S. italica* MADS-box genes during fruit development. (**A**) qRT-PCR was used to detect the expression patterns of 12 *S. italica* MADS-box genes in peel and fruit before, during and after grain filling. Error bars were obtained from three measurements. Lowercase letter above the bar indicates significant difference (α = 0.05, LSD) among treatments. (**B**) Positive number: positively correlated; negative number: negatively correlated. Red numbers indicate a significant correlation at the 0.05 level.

Foxtail millet fruit are rich in calcium, dietary fiber, polyphenols, fats, proteins and other nutrients^17,18^. Tissue-specific expression of *Si*MADS-box genes was also found to be high in fruit. Therefore, we determined the expression level of *Si*MADS-box genes in peel and fruit before, during and after grain-filling (Fig. 8A). Two genes (*SiMADS02* and *SiMADS17*) showed very low expression in the peel. Five genes (*SiMADS28*, *SiMADS33*, *SiMADS46*, *SiMADS52*, *SiMADS60*) were highly expressed in the peel, mainly at the early filling stage. M-type *SiMADS60* also exhibited high expression in the middle filling stage, and M-type *SiMADS28* in the late filling stage.

Compared to the peel, all genes were highly expressed in the fruit, eight genes in particular (*SiMADS12*, *SiMADS26*, *SiMADS28*, *SiMADS33*, *SiMADS37*, *SiMADS46*, *SiMADS60*, *SiMADS67*). Among these genes, there were differences in expression for those belonging to the MIKC-type and M-type. MIKC-type genes were mainly highly expressed in the early filling stage (*SiMADS12*, *SiMADS26*, *SiMADS46*) and middle filling stage (*SiMADS33*, *SiMADS37*), whereas M-type genes *SiMADS28* and *SiMADS60* were mainly highly expressed in the late filling stage.

We also studied the correlation between *Si*MADS-box gene-expression patterns in peel and fruit at different filling stages and found both positive and negative correlations (Fig. 8B). For example, the MIKC-type *SiMADS12* and *SiMADS26*, which were highly expressed in the early stage of fruit-filling, were significantly negatively correlated with the M-type *SiMADS28* and *SiMADS60*, which were highly expressed in the late stage of grain-filling. This also revealed that there were differences in expression of *Si*MADS-box genes of the MIKC-type and M-type. There were also some similarities, for example, a significant positive correlation between M-type *SiMADS17* and *SiMADS78* expression, which were also significantly positively correlated with expression of the MIKC-type genes *SiMADS33* and *SiMADS37*, which were highly expressed in fruit at the middle stage of filling.

### Expression patterns of *Si*MADS-box genes in response to different abiotic stresses

**Figure 9:**
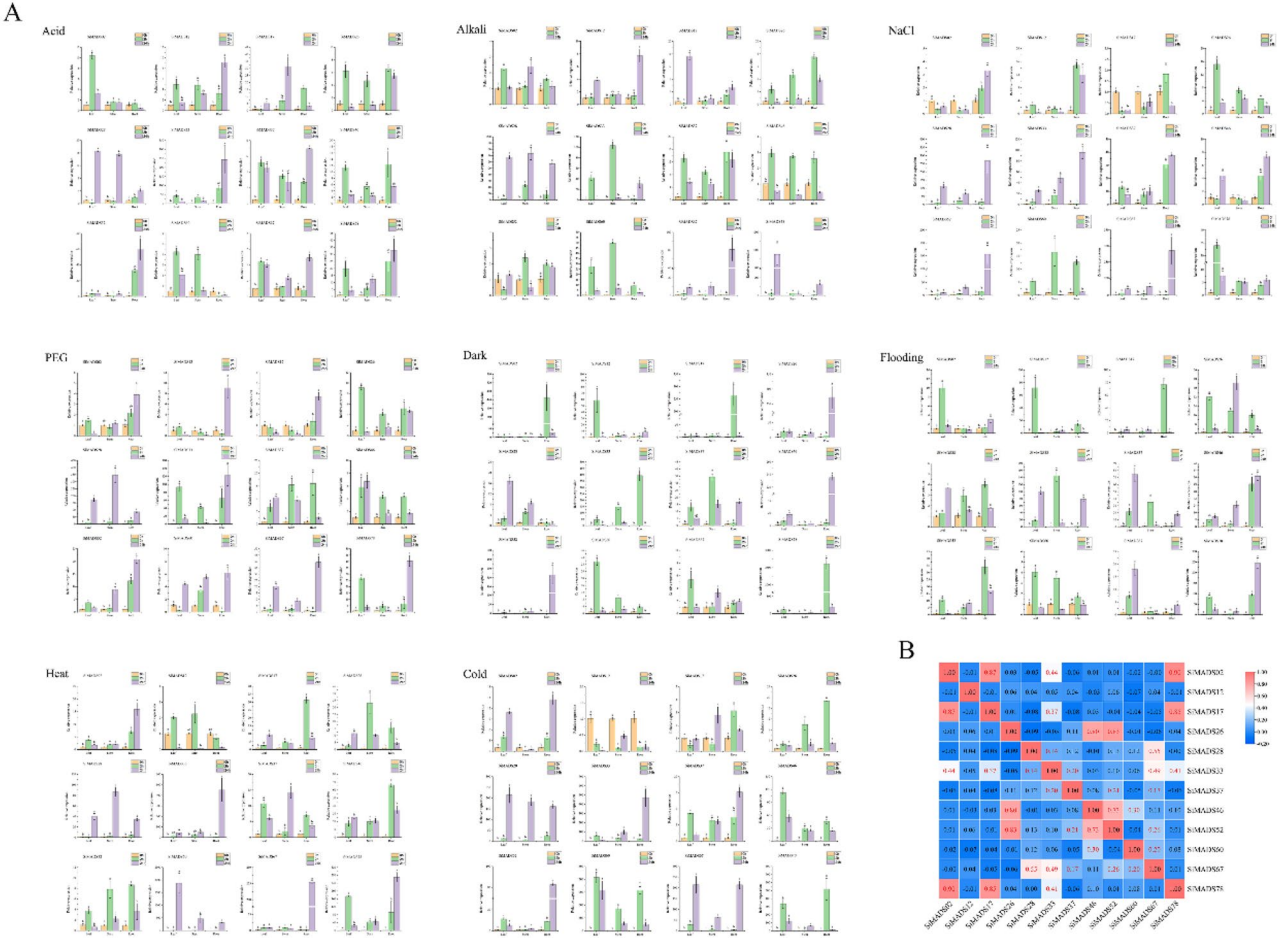
Gene expression of 12 *S. italica* MADS-box genes in plants subjected to abiotic stresses (acid, alkali, NaCl, PEG, dark, flooding, heat, cold) at the seedling stage. (**A**) qRT-PCR was used to detect the expression patterns of 12 *S. italica* MADS-box genes in roots, stems and leaves at different times. Error bars were obtained from three measurements. Lowercase letter above the bar indicates significant difference (α = 0.05, LSD) among treatments. (**B**) Positive number: positively correlated; negative number: negatively correlated. Red numbers indicate a significant correlation at the 0.05 level.

To determine whether the expression of *Si*MADS-box genes is affected by different abiotic stresses, we analyzed the expression of 12 *Si*MADS-box genes under eight kinds of abiotic stress: acid, alkali, NaCl, polyethylene glycol (PEG), flooding, dark, heat and cold. We used qRT-PCR to analyze the expression patterns of these 12 genes in leaves, stems and roots under the different treatments. Some *Si*MADS-box genes were significantly induced, whereas others were suppressed (Fig. 9A). For example, *SiMADS33* and *SiMADS78* were significantly induced under several abiotic stresses (acid, alkali, dark, heat and cold treatments). In addition, some genes showed different patterns under different treatments, such as *SiMADS28*, the expression of which was significantly upregulated under alkali, salt and PEG treatments, but changed little under flooding. *SiMADS60* expression was significantly upregulated in both heat and cold treatments, but its expression also did not change significantly under flooding. *SiMADS33* and *SiMADS67* tended to be highly expressed in roots, *SiMADS78* tended to be expressed in roots and leaves, and expression of *SiMADS28, SiMADS37* and *SiMADS60* was significantly upregulated in roots, stems and leaves. In general, the expression levels of genes that tended to be expressed in roots peaked after 24 h of stress, whereas the expression levels of those that tended to be expressed in stems and leaves could be detected after 2 h of stress. A correlation between *Si*MADS-box gene-expression patterns under stress was also observed (Fig. 9B). Most *Si*MADS-box genes were positively correlated. For example, there was a significant positive correlation between the expressions of *SiMADS33* and *SiMADS28*, *SiMADS67* and *SiMADS78*, and between *SiMADS02* and *SiMADS17* (*P* < 0.05).

## Discussion

### Characteristics of *Si*MADS-box genes

We analyzed 89 SiMADS-box proteins with molecular weight ranging from 6.78 to 53.18 kDa and pI ranging from 4.41 to 11.39. Several alkaline residue-rich fragments in the MADS domain contain nuclear localization signals^38^, so the MADS-box proteins are thought to be located in the nucleus^39^. Many MADS-box genes have been subcellularly localized, and indeed, most of them are located in the nucleus, such as *AGL15*^40^, *AGL24*^41^, *AGL61*^42^, *AGL80*^43^ in *A. thaliana* and *OsMADS22*, *OsMADS47*, *OsMADS50*^44^ in rice. In this study, most of the *Si*MADS-box genes were predicted to be located in the nucleus, as expected. The *Si*MADS-box genes were divided into M-type (37) and MIKC-type (52). It is interesting that no *Si*MADS-box genes were included in the FLC-like subfamily, which is associated with vernalization^45^. This may be because foxtail millet does not have a vernalization requirement, so this branch may have been lost in this species. Gene-structure analysis showed a great difference in the average number of introns between the two subfamilies, with the average number of introns in the MIKC-type subfamily being much larger than that in the M-type subfamily. This is similar to the distribution of introns in rice^1^ and *A. thaliana*^25^, and indicates that the two types of *Si*MADS-box genes have different evolutionary paths, possibly due to their different tendencies to lose or gain introns during differentiation^25^. We speculate that in the evolution of foxtail millet MADS genes, under the pressure of natural selection, M-type MADS genes may tend to lose a large number of introns. As a result, the number of introns in the M-type MADS gene is very small or no intron, which suggests that the M-type MADS gene may have played a role in the evolution of foxtail millet. As for why the M-type and MIKC_type MADS genes produced different evolutionary paths, it is currently unclear, which is also worthy of in-depth research in the future. In addition, the MIKC and M subfamilies have their own unique conserved motif sequences, implying that their functions may also differ. A phylogenetic tree, constructed with MADS genes of other species, showed that the motif distribution in the MIKC subfamily was more conserved than that in the M-type MADS genes. In the M-type phylogenetic tree, the subfamily not only included the most *Si*MADS-box members (19), but it also had a unique conserved motif (motif 8), but lacked motif 7–1. In the MIKC-type phylogenetic tree, subfamily f was relatively unique, and new motifs 9 and 10 appeared. The unique motifs in these subgroups may endow the subfamily with special physiological functions. On the other hand, similar amino acid structures in each subfamily suggest similar physiological functions.

### Evolution of *Si*MADS-box genes

Most of the duplications of foxtail millet genes stemmed from whole genome duplication (WGD) events that are common to all Gramineae^19^. We analyzed the duplication events in foxtail millet MADS-box genes and found only one pair of tandem repeats (*SiMADS69* and *SiMADS70*) in the MIKC-type genes, but 11 pairs of duplicated segment genes. Among the *Si*MADS-box genes with segmental duplications, only 1 pair belonged to the M-type, and the other 10 pairs belonged to the MIKC-type. Among these repetitive events, the TM3-like subfamily had three pairs. This is similar to the situation of segmental duplications in rice^1^, that is, MIKC-type genes account for the vast majority of the segmental duplications. Therefore, some *Si*MADS-box genes may have been generated by gene-replication events, in which the amplification of MIKC-type genes is the main force driving the amplification of the number of *Si*MADS-box genes, and the number of MIKC-type genes (52) is thus much larger than that of the M-type genes (37). In addition, collinearity analysis with other species showed the highest collinearity for both M-type and MIKC-type genes with maize, and collinearity with monocotyledons was much higher than that with dicotyledons. Some of the MIKC genes showed collinearity with both monocots and dicots. Therefore, we speculate that the differentiation of the MIKC subfamily occurred earlier than that of monocotyledons and dicotyledons. In addition, the genes may be so fundamental to the different plants that they cannot be lost. However, these genes included only two duplicated genes, suggesting that the other nine duplicated genes were formed after the differentiation of monocotyledons and dicotyledons. We found four genes that were only collinear with maize, and these genes may be characteristic of C4 plants. These results were expected, because WGD of foxtail millet occurred before the separation of sorghum and maize^19^. Foxtail millet, as a monocotyledonous C4 crop^46,47^, is more closely related to maize and monocotyledons. Naturally, it has the most collinear genes with maize, which is also a C4 crop, and should have more collinear genes with other monocotyledons than dicotyledons.

### Temporal and spatial expression of *Si*MADS-box genes

The expression of MADS-box genes has been investigated in the tissues of different species, for example, in root, leaf and inflorescence of *A. thaliana*^25^, and in roots, stems, leaves and flower organs of Brachypodium distachyon^48^. In rice, most MADS-box genes are specifically expressed in the panicle and seed^1^. In this study, we found expression of *Si*MADS-box genes in various organs and to varying degrees. Most of the genes were highly expressed in fruit, especially MIKC-type *SiMADS33* and *SiMADS37*. The correlation of genes' expression in different tissues was also explored. Expression of M-type and MIKC-type genes was more positively correlated in certain tissues, indicating their similar functions in these tissue parts.

Furthermore, the expression of *Si*MADS-box genes in the peel and fruit before, during and after grain-filling was discussed. Different genes were involved in the whole process of fruit development during grain-filling, that is, genes were highly expressed before, during or after grain filling. However, M-type genes *SiMADS28* and *SiMADS60* tended to be highly expressed in the late filling period, whereas MIKC-type genes tended to be highly expressed in the early filling period (*SiMADS12*, *SiMADS26*, *SiMADS46*) and middle filling period (*SiMADS33*, *SiMADS37*). There were not only significant positive correlations, but also significant negative correlations between MIKC-type and M-type genes in the early, middle and late stages of grain-filling. These results indicate that the functions of MIKC-type and M-type genes in the process of grain-filling can be similar or different. Predicted protein interactions indicated that *Si*MADS46 protein interacts with *Si*MADS26, *Si*MADS52 and *Si*MADS60 proteins (Fig. 3B). The correlation analysis results of the relative expression levels of 12 genes also showed significant correlations between *SiMADS46* and *SiMADS26*, *SiMADS52* and *SiMADS60*. These results also confirm the reliability of the predicted protein-interaction results, and suggest that these proteins may indeed interact.

Previous studies have found that *OsMADS22* (LOC_Os02g52340) and *OsMADS55* (LOC_Os06g11330) are highly expressed in the stem, significantly inhibiting stem elongation in coordination with negative regulation of brassinosteroid content^49,50^. However, the homologous gene *SiMADS12* was not highly expressed in the stem. *OsMADS29* (LOC_Os02g07430) plays an important role in the development of rice seeds^51^, and its homologous gene *SiMADS2* was also highly expressed at the early stage of seed filling, suggesting that *SiMADS2* may also play a crucial role in the development of foxtail millet seeds.

### Response of *Si*MADS-box genes to abiotic stress

Studies have been more focused on exploring the role of MADS-box genes in floral organ development, and less on exploring their response to various abiotic stresses^48,52^ However, some studies have found that the response of M-type MADS-box genes to abiotic stress is very important^48^. We found some genes to be significantly induced under stress, such as *SiMADS33* and *SiMADS78* under acid, alkali, dark, heat and cold treatments. *SiMADS33* and *SiMADS78* may be key genes in stress tolerance, warranting further study. Some genes showed different expression patterns under different treatments, such as *SiMADS28* which was significantly upregulated under alkali, salt and PEG treatments, but changed little under flooding. Some genes may be greatly affected by temperature stress, such as the significant increase of *SiMADS60* expression under cold and heat stress treatments. Expression tendencies also differed, for example, *SiMADS33* and *SiMADS67* tended to be highly expressed in roots, whereas *SiMADS78* tended to be expressed in roots and leaves. The response time of the different genes to the stresses also differed. It was frequently found that those genes which were highly expressed in stems and leaves could respond to stress quickly, with significant changes in expression detected after 2 h of the stress. However, the response time of genes that were highly expressed in roots was relatively slow, reaching peak expression after 24 h of stress. The expression of *Si*MADS-box genes under the stress treatments was mostly positively correlated, suggesting interactions between genes, so as to jointly deal with the adverse effects of the abiotic stress on the plants. Protein-interaction prediction showed that the three MIKC proteins (*Si*MADS26, *Si*MADS46, *Si*MADS52) interacted, and that *Si*MADS46 also interacted with the M-type protein *Si*MADS60 (Fig. 3B). In addition, correlation analysis of the relative expression of the 12 genes under eight abiotic stresses also showed a significant positive correlation among *SiMADS26*, *SiMADS46*, and *SiMADS52*. *SiMADS46* and *SiMADS60* were also significantly positively correlated. Therefore, we speculate that there is interaction between these proteins under external environmental stress, which makes the plant better through the stress period.

In addition, other genes may also have important biological functions, such as *SiMADS52*, which is highly expressed in stems. Its collinearly related gene *AGAMOUS* (*AT4G18960*) regulates sepal senescence by promoting the production of jasmonic acid^53^. In addition, *AGAMOUS-like15* (*AGL15*) and *AGL18*^54,55^ can delay sepal senescence and anther ablation in *A. thaliana*, thereby controlling the time of flower senescence. The homologous gene *OsMADS58* (LOC_Os05g11414) plays a key role in regulating flower meristem decisions, and *OsMADS3* (LOC_Os01g10504) plays an important role in regulating stamen characteristics^56^. This suggests that *SiMADS52* may also have a similar function, warranting further study. Previous studies have found that *OsMADS2* (LOC_Os01g66030) and *OsMADS4* (LOC_Os05g34940) play an important role in style and stamen development^57^, and their homolog *SiMADS26* may play a similar major role in this development.

## Materials and methods

### Plant materials, growth conditions, and abiotic stress treatments in foxtail millet

The test material in this study was the foxtail millet *Setaria italica* cv. Yugu 1, typical to northern China, and planted in a greenhouse. We obtained samples of roots, stems, leaves, peels and fruit in the middle grain-filling stage, and of peels and fruit in the early and late filling stages. All samples were taken from five plants under the same growing conditions, quickly frozen in liquid nitrogen, and stored at − 80 °C. The expression levels of 12 *Si*MADS-box genes were detected. In addition, we carried out stress treatments on foxtail millet plants at the seedling stage (28 days), including salt (5% NaCl), acid (0.1 mol/L HCl), alkali (0.2 M NaOH), darkness (complete shading), flooding (whole plant), drought (10% PEG6000), heat (40 °C), and cold (4 °C). Five repeats were carried out for each stress treatment, and samples were collected at 0, 2 and 24 h for qRT-PCR analysis.

### Total RNA extraction, cDNA reverse transcription and qRT-PCR analysis

Total RNA was extracted by RNA extraction kit (TaKaRa Bio). The qRT-PCR primers (Table S6) were designed using Primer 5.0 software. In this experiment, the actin gene (*Si001873mg*)^58^ was used as an internal control (http://www.pantherdb.org/genes/gene.do?acc=SETIT%7CEnsemblGenome%3DSETIT_001873mg%7CUniProtKB%3DK3XJ00). Standard qRT-PCR with SYBR Premix Ex Taq II (TaKaRa Bio) was repeated at least three times on a CFX96 Real-Time PCR System (Bio-Rad). The qRT-PCR data were analyzed by the 2^−(ΔΔCt)^ method.

### Genome-wide Identification of MADS-box genes in foxtail millet

The *Setaria italica* genome was downloaded from EnsemblPlants (https://plants.ensembl.org/info/website/ftp/index.html). The MADS-box protein sequences of *A. thaliana* (https://www.Arabidopsis.org/) and rice (http://Rice.plantbiology.msu.edu/) were downloaded separately. First, the whole foxtail millet genome was aligned with AtMADS and OsMADS protein sequences (score value ≥ 100 and e-value ≤ 1e^−10^), yielding the candidate MADS-box genes. Second, the hidden Markov model (HMM) of the M-type SRF-TF domain (PF00319), MIKC-type SRF-TF domain (PF00319) and MIKC-type K-box domain (PF01486) was downloaded from the Pfam database (http://pfam.xfam.org/)^59^, and HMMER 3.0 software (with default parameters) (http://HMMER.org/)^60^ was used to search for MADS-box proteins. The obtained sequences were further verified by SMART tool (http://SMART.emblheidelberg.de/)^61^ to identify a putative MADS domain. Finally, the sequences of 89 MADS-box proteins were obtained. Then their length, molecular weight and pI were determined on the ExPasy website (https://web.expasy.org/compute_pi/). Subcellular localization of MADS-box proteins was predicted by WoLFPSORT (https://wolfpsort.hgc.jp/)^62^.

### Phylogenetic analysis and classification of the *Si*MADS-box gene family

According to the classification of MADS-box genes in rice and *A. thaliana* and the *Si*MADS-box domain, the identified *Si*MADS-box genes were divided into groups. Phylogenetic trees were constructed using protein sequences from *A. thaliana*, *O. sativa*, *Brassica napus*, *Zea mays*, *Fagopyrum tataricum*, and *Brachypodium distachyon*) downloaded from the UniProt database (https://www.UniProt.org). We used MUSCLE sequence alignment for the protein sequences, and then constructed the ML phylogenetic tree with IQ-tree wrapper, and bootstrap number set to 1000.

### Chromosomal distribution and gene duplication of *Si*MADS-box genes

We use Circos to obtain information on the genes' physical location in the foxtail millet genome and localized all *Si*MADS-box genes to the chromosomes. The Multiple Collinearity Scan toolkit X (MCScanX) was used with default parameters to scan the collinearity of *Si*MADS-box genes and then analyze gene-duplication events. We used a Double Synteny Plotter to analyze the homology of *Si*MADS-box genes among species^63^.

### Gene structure, conserved motif analysis and protein-interaction prediction

The structural map of *Si*MADS-box genes was constructed by sequence alignment between the CDS and the corresponding genomic DNA sequence. The online MEME tool (http://meme-suite.org/tools/meme)^64^ was used to analyze the full-length conserved motifs of the *Si*MADS-box family of proteins, and the maximum conservative motif search value was set to 10. SiMADS-box protein interactions were predicted using STRING (https://string-preview.org/).

### Statistical analysis

Analysis of variance (ANOVA) was performed with JMP6.0 software (SAS Institute), and least significant difference (LSD) was used for comparisons at the 0.05 and 0.01 levels. The histogram was drawn with OriginPro2019b software (OriginLab).

### Ethics approval and consent to participate

The foxtail millet accession (Yugu 1) was supplied by Professor Jianping Cheng of Guizhou University. These plant materials are widely used all over the world and no permits are required for the collection of plant samples. This article does not contain any studies with human participants or animals performed by the authors. The methods were carried out in accordance with the relevant guidelines and regulations. We confirm that all experimental protocols were approved by Guizhou University.

## Supplementary Information

Supplementary Information.

## Data Availability

Information on the entire *Setaria italica* genome sequence was from the Ensembl Genomes website (http://ensemblgenomes.org/). The *Setaria italica* materials (Yugu 1) used in the experiments were supplied by Prof. Jianping Cheng of Guizhou University. The datasets supporting the conclusions of this article are included in the article and its Supplementary Material.
